# A Comprehensive Overview on the Production of Vaccines in Plant-Based Expression Systems and the Scope of Plant Biotechnology to Combat against SARS-CoV-2 Virus Pandemics

**DOI:** 10.3390/plants10061213

**Published:** 2021-06-15

**Authors:** Manu Kumar, Nisha Kumari, Nishant Thakur, Shashi Kant Bhatia, Ganesh Dattatraya Saratale, Gajanan Ghodake, Bhupendra M. Mistry, Hemasundar Alavilli, D. S. Kishor, Xueshi Du, Sang-Min Chung

**Affiliations:** 1 Department of Life Science, College of Life Science and Biotechnology, Dongguk University, Seoul 10326, Korea; manukumar007@gmail.com (M.K.); kishoreflmes@gmail.com (D.S.K.); duxueshii@gmail.com (X.D.); 2 Department of Radiology, Seoul National University Hospital, Seoul National University College of Medicine, Seoul 03080, Korea; nishak.chambyal88@gmail.com; 3 Department of Hospital Pathology, Yeouido St. Mary’s Hospital, College of Medicine, The Catholic University of Korea, 10, 63-ro, Yeongdeungpo-gu, Seoul 07345, Korea; nishantbiotech2014@gmail.com; 4 Department of Biological Engineering, College of Engineering, Konkuk University, 1 Hwayang-dong, Gwangjin-gu, Seoul 05029, Korea; shashibiotechhpu@gmail.com; 5 Department of Food Science and Biotechnology, Dongguk University, Seoul 10326, Korea; gdsaratale@gmail.com (G.D.S.); bhupendra.mistry84@gmail.com (B.M.M.); 6 Department of Biological and Environmental Science, Dongguk University, Seoul 10326, Korea; ghodakegs@gmail.com; 7 Department of Biochemistry and Molecular Biology, College of Medicine, Korea University, Seoul 02841, Korea; alavilli.sundar@gmail.com

**Keywords:** SARS-CoV-2 virus, COVID-19 vaccine, bio-farming, respiratory disorder, vaccine

## Abstract

Many pathogenic viral pandemics have caused threats to global health; the COVID-19 pandemic is the latest. Its transmission is growing exponentially all around the globe, putting constraints on the health system worldwide. A novel coronavirus, severe acute respiratory syndrome coronavirus-2 (SARS-CoV-2), causes this pandemic. Many candidate vaccines are available at this time for COVID-19, and there is a massive international race underway to procure as many vaccines as possible for each country. However, due to heavy global demand, there are strains in global vaccine production. The use of a plant biotechnology-based expression system for vaccine production also represents one part of this international effort, which is to develop plant-based heterologous expression systems, virus-like particles (VLPs)-vaccines, antiviral drugs, and a rapid supply of antigen-antibodies for detecting kits and plant origin bioactive compounds that boost the immunity and provide tolerance to fight against the virus infection. This review will look at the plant biotechnology platform that can provide the best fight against this global pandemic.

## 1. Introduction

In late 2019, a potentially lethal outbreak of novel coronavirus (SARS-Cov-2) with the fatal respiratory syndrome was reported in Wuhan, China [1]. This outbreak has created a pandemic all over the world. Up to June 2021 (sixteen months after its emergence), it has caused more than 175,306,598 cases of infections and more than 3,792,777 deaths worldwide, affecting 223 countries (WHO) [2]. COVID-19 has a higher mortality rate (~2.2%) and transmissibility than the influenza A virus subtype H1N1 pandemic, which has a mortality rate of around 0.02%. Coronaviruses are single-stranded RNA virus that is grouped into four categories: α-CoVs, β-CoVs, γ-CoVs, and δ-CoVs.

Governments are trying to control this outbreak by emergency containment and rapid testing. These measures will slow the infection rate, reduce the mortality rate, and prevent the healthcare system from collapsing. In addition, it will allow researchers to have enough time to develop fast testing kits and treatments that limit the infection and the candidate vaccine to immunize the population. Researchers working on plant-based vaccines can also play a crucial role during this crucial time by using their knowledge and platform to develop a reagent as early as possible compared to months and years based on a cell-based platform.

## 2. Progress in Prophylactic and Therapeutic Treatments against COVID-19

### 2.1. Candidate Vaccine for COVID-19

For COVID-19, there is no specific treatment at this stage. Several technological gaps exist for SARS-CoV-2 virus understanding, as it is still an early stage for this pathogen. Currently, 102 candidate vaccines are in clinical trials, and 185 candidate vaccines are in preclinical trials [3]. Thirty-one percent of candidate vaccines are based on protein subunit platforms (Table 1). WHO issued an emergency use listing (EULs) for the mRNA-based Pfizer COVID-19 vaccine (BNT162b2) on 31 December 2020. On 15 February 2021, WHO again issued EULs for two versions of the viral vector-based AstraZeneca/Oxford COVID-19 vaccine, manufactured by the Serum Institute of India and SKBio [3]. There are more than 15 other candidate vaccines that await WHO listing. An international effort is ongoing for vaccine procurements. Simultaneously, the determination of the efficacy of preexisting antiviral drugs, such as Remdesivir, Nafamostat, and camostat, is taking place [4,5,6]. Receptors are essential for the attachment of any virus, and by blocking the receptor, virus attachment can be inhibited. It is reported that losartan (an angiotensin receptor 1 blockers) might have tentative SARS-CoV-2 therapeutics value since angiotensin-converting enzyme 2 (ACE2) likely to serves as the binding site for the SARS-CoV-2 [7,8]. Transfusing plasma from individuals recovered from COVID-19 infection also shows promise as plasma contains neutralizing antibodies for SARS-CoV-2 [9]. For the control and timely eradication of infectious diseases, vaccination is the most potent weapon. Since transmission of SARS-CoV-2 is very high, there is an urgency to develop the vaccine and eradicate this virus. The simplest way to generate a candidate vaccine lies in the technology where an inactivated virus can be used for vaccine production [10]. The live-attenuated virus vaccine is another possible approach where these vaccines lost their pathogenic properties and caused only a mild infection upon injection [11].

An earlier candidate inactivated virus vaccine for MERS-CoV and SARS-CoV-1 has helped neutralize the virus [12,13,14]. Another path is to construct a recombinant live attenuated vaccine virus that can protect from SARS-CoV-2 and respiratory syncytial virus. It has already been reported in the case of the influenza virus vaccine [15]. Another alternate can be an adenovirus-based vaccine that prevented pneumonia from SARS coronavirus and stimulated a good immune response in macaques [13]. Adenoviruses are vectors used to deliver vaccine antigens to the target host tissues and are being tested in several gene therapies and vaccine studies [16]. Several leading adenovirus-based vaccine candidates are in advanced phases of clinical trials (Table 2), such as ChAdOx1-S-(AZD1222) (Covishield) (AstraZeneca + University of Oxford), Recombinant novel coronavirus vaccine (Adenovirus type 5 vector) (CanSino Biological Inc./Beijing Institute of Biotechnology), Gam-COVID-Vac Adeno-based (rAd26-S + rAd5-S) (Gamaleya Research Institute; Health Ministry of the Russian Federation), Ad26.COV2.S (Janssen Pharmaceutical). Another approach would be a DNA-based vaccine, where deoxyribose nucleic acid (DNA) codes for specific proteins (antigens) from a pathogen are injected into the body and taken up by cells and generate an immune response. The nCov vaccine from Zydus Cadila is a DNA-based vaccine in the advanced clinical trials phase. Even though many countries are rushing towards generating vaccines for SARS-CoV-2, safety regulation guarantees should not be ignored [17]. Most of the candidate vaccines in advanced clinical trials have good efficacy data (Table 2).

**Figure 1:**
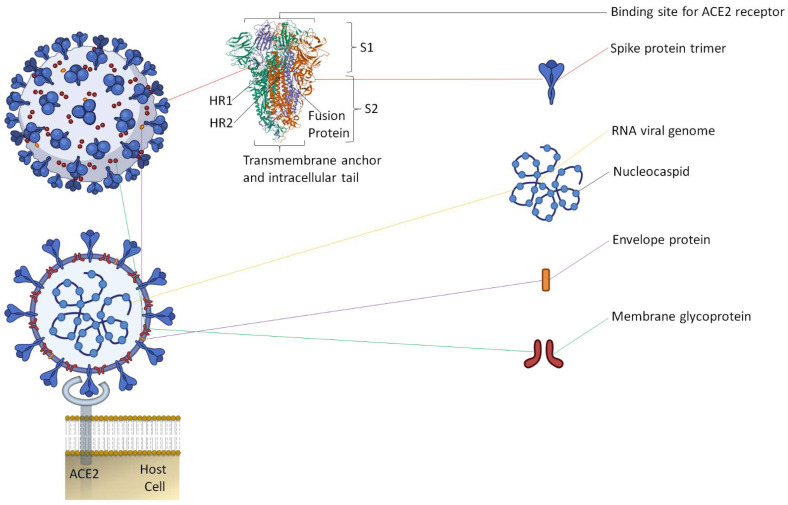
The representative two-dimensional structure of Severe Acute Respiratory Syndrome Coronavirus 2 (SARS-CoV-2) shows the trimeric spike protein’s prominent position. The virus is constituted by an envelope membrane that is associated with the structural proteins, such as spike protein trimer, which mediates binding to the host cell ACE2 receptors and considered a vital target for the activation of a primary defense mechanism by the induction of antibodies that are capable of neutralizing the virus. 2-D structure (PDB ID: 6XLU) of spike protein has two subunits, S1 and S2. S2 subunit has two main domain, HR1 (912–984 aa) and HR2 (1163–1213 aa), along with fusion protein that contains the significant parts of HR1 (residues 910–988) and HR2 (residues 1162–1206); a membrane glycoprotein, which is essential to generate the virus; and the envelope protein, which adheres to the membrane glycoprotein to form the viral envelope. The viral structure also comprises a nucleocaspid protein that, along with the RNA genome, produces the nucleocaspid. The figure includes some images from Biorender (https://biorender.com/, accessed on 12 June 2021).

Along with the inactivated vaccines approach, an alternative method should be explored for candidate vaccines. Since spike protein trimers are the primary binding sites of the ACE2 receptor of a host cell, it makes this protein an easy target for antibody neutralization (Figure 1) [18,19].

Another approach would be RNA-based vaccines. Here, instead of the standard vaccines where viral proteins are used to immunize, the messenger RNA vaccine will provide a synthetic mRNA of the virus, which the host body will use to produce an immune response [20,21,22,23,24,25,26]. The most significant advantage of the RNA vaccines is that they are translated in the cytosol, so there is no need for the RNA to enter the cell nucleus, and the risk of being integrated into the host genome is averted [27]. RNA-based candidate vaccine that are in most advanced phased of clinical trials are mRNA-1273 (Moderna + National Institute of Allergy and Infectious Diseases), BNT162b2 (Pfizer/BioNTech + Fosun Pharma), CVnCoV (CureVac AG) [28,29] (Table 2). An inactivated virus vaccine is also another approach, where a killed version of the virus is used. Inactivated vaccines usually cannot provide immunity as much as live vaccines. Several booster doses over a while are required to get uninterrupted immunity against particular diseases. Several vaccine candidates used Inactivated virus such as CoronaVac (Sinovac Research), Inactivated SARS-CoV-2 vaccine (Sinopharm + China National Biotec Group Co + Wuhan Institute of Biological Products), Sinopharm + China National Biotec Group Co + Beijing Institute of Biological Products, Institute of Medical Biology + Chinese Academy of Medical Sciences, QazCovid-in^®^ (Research Institute for Biological Safety Problems, Rep of Kazakhstan), BBV152 (Bharat Biotech International Limited). There are other candidate vaccines also that used protein subunit of SARS-CoV-2. Protein-based subunit vaccine presents an antigen to the immune system without viral particles using a specific pathogen protein (Table 2). These candidate vaccines have shown well-documented immunogenicity in the preliminary studies [30]. Using bioinformatics for the detailed analysis of sequence analysis is another approach to predict immune response for SARS-CoV-2 [31]. SARS-CoV-2 structure’s analysis will help in understanding the response strategy towards this virus. There are reports about the structure, function, and antigenicity predictions of the SARS-CoV-2 spike glycoprotein [19]. These predictions will be important for the designing of vaccines and inhibitors of viral receptors. Another study reports that the crystal structure of SARS-CoV-2 main protease provides a basis for the design of improved α-ketoamide inhibitors [32]. These recent studies will help in the ongoing effort to produce effective vaccines, but in parallel to this vaccine development, a reliable alternative platform that can provide rapid and large-scale vaccine production is needed. The platform must be designed to keep in mind low cost, easy distribution, and special campaigns in poor and developing countries.

### 2.2. Promising Adjuvants Used for the Development of COVID-19 Vaccines

Adjuvants are critical components of some inactivated and subunit vaccines because they help in boosting the specific immune responses against vaccine antigens [33,34]. In the last decades, many materials have been tried and tested as adjuvants. Examples include bacterial metabolites [35,36], mineral oil/surfactant with immune-stimulant [37], microparticles [38,39], nucleic acids [40], liposomes [41,42], and polysaccharide [43]. However, only aluminum-based adjuvants continue to be used worldwide [44].

Alum (aluminum hydroxide) is one of the most commonly used adjuvants. Its mechanism of action is complex. So far, multiple hypotheses have come to light to explain its mode of action [45]. It forms a depot at the injection site allowing a slow release of antigen; it prolongs the interaction time between antigen-presenting cells (APCs) and antigen; further, it converts soluble antigens into readily phagocytosed particulate forms [46]. Aluminum hydroxide directly stimulates monocytes at the cellular level to produce pro-inflammatory cytokines activating T cells. Most of the COVID-19 vaccines are using aluminum hydroxide as their adjuvant (Table 2).

Another aluminum adjuvant commonly used in vaccine productions is aluminum phosphate (AlPO4). It also stimulates the immune response against antigens. They are required for the efficacy and optimal immunogenicity of many vaccines. Onto the surface of adjuvants, different antigens adsorb to different extents and can undergo structural changes that may destabilize or stabilize the antigens. Upon adjuvant action, bovine serum albumin, lysozyme, and ovalbumin experience a decrease in the unfolding temperature [47]. In another study, antigen protein for tuberculosis vaccine candidates became more stable upon adsorption onto a different type of adjuvant [48]. Janssen Pharmaceutical using AlPO4 as an adjuvant for their COVID-19 vaccine Ad26.COV2.S (Table 2).

Another adjuvant CV8102 is a TLR 7/8 agonist and RIG I pathway activator to enhance the immunogenicity of poorly immunogenic antigens. It is an RNA-based adjuvant (RNAdjuvant^®^, CureVac AG) [49,50]. It consists of uncapped, non-coding, PolyU repeats-containing single-stranded RNA with a 5-triphosphate modification complexed with a polymeric carrier and a small arginine-rich disulfide-cross-linked cationic peptide (CR12C) [50,51]. CureVac AG RNA based vaccine CVnCoV using CV8102 as an adjuvant.

Another important adjuvant, AS03, has a polysorbate 80, surfactant, two biodegradable oils, squalene, and α-tocopherol in phosphate-buffered saline as the aqueous carrier [52]. These adjuvant capabilities derive from the α-tocopherol, and oil-in-water emulsion phase, for which immunostimulatory properties have been described [53]. After a cascade of reactions, the AS03 adjuvant enhances adaptive immune responses to the vaccine antigen [52,54]. The Sanofi Pasteur protein subunit vaccine VAT00002 uses the GSK adjuvant AS03.

The Novavax protein subunit vaccine uses Matrix-M as an adjuvant (Table 2). It is made of *Quillaja saponins* formulated with phospholipids and cholesterol into nanoparticles and is known to augment Th1 and Th2, inducing antibodies of multiple subclasses that enhance immune cell trafficking and allow antigen dose-sparing [55,56,57,58,59,60].

## 3. Plant Biotechnology-Based Vaccines and Bio-Farming?

Transgenic plant from a genetic engineering approach provides a perfect platform for the manufacturing of large-scale biopharmaceuticals. In the last three decades, these plants have been used widely for the production of biopharmaceuticals. This approach has produced a wide range of biopharmaceuticals, such as cytokines, growth factors, antibodies, and vaccines [61].

The production of antibodies in transgenic tobacco plants was reported by Hiatt et al. [62]. It was the first example of bio-farming, where the aim is to recover and use only protein products instead of the whole plant [63,64,65]. Recombinant human serum albumin is produced in transgenic potato and tobacco plants by overexpressing the human serum albumin gene [66,67]. These path-breaking studies open the flood gates for bio-farming in plants [68]. These plant-based viral expression systems’ main advantages are to avoid human pathogens replication, easy synthesis of complex proteins, and utilization of simple bioreactors [69,70].

### 3.1. Strategies for the Production of Recombinant Proteins in Plant-Based Expression Systems

In plant-based expression systems, there are three main approaches for recombinant protein production [28]: (1) by developing transgenic plants carrying stably integrated transgenes [63]; (2) using cell-culture-based systems equivalent to microbial, insect cell, and mammalian systems; and (3) by transient expression of foreign genes in plant tissues transformed by either a viral infection or agroinfection [66,71,72]. For a foreign gene’s proper function in a host cell, the gene must replicate into many copies in the cell’s nucleus; hence, these transient expressions happen at the nucleus. Currently, transiently transformed plants at nuclear or chloroplast are used to produce expressed recombinant protein. In Table 3, the expression method is summarized.

The most common approach for expressing a transgene in the plant includes transgene insertion in the genome, and agroinfection by *Agrobacterium*-mediated transformation is a widely used method since these bacteria can transfer large insert with a highly efficient low number of insertion. It is crucial for stable transformation. The limitation of this method is that gene insertion is random, called a positional effect. Due to this, the expression level might have variations depends on the event, and sometimes it affects the expression of the endogenous gene. However, It has more pros than cons, and with the emergence of new technology, these limitations can be overcome by site-directed insertion by several mechanisms such as genome editing by zinc finger nucleases, TALENs, and CRISPR/Cas9 System, etc. [78].

Site-directed insertion of the foreign DNA into the chloroplast genome resulted from homologous recombination. High protein yield is the plus point of this technology, directly resulting from the transgene’s high copy number. There is no report of silencing events and position effects in this method. Moreover, a single transformation event can produce an abundance of proteins [79,80]. More detail about plastid-based expression can be found in the given articles [79,80,81].

Heterologous expression of a protein in plants by delivering virus-based vectors to their target via agroinfection is another approach. This method has a dependence on the DNA/RNA replication mechanism, untranslated regions (UTRs), and promoter efficiency in plant viruses. This method can produce high-yield proteins. For example, GFP yields as high as 5 mg/g of fresh weight tissue (FWT) are reported [82]. 

The comoviruses, geminiviruses, potexviruses, tobamoviruses, and tobraviruses were used as a platform for efficient transient expression in plants [83]. In this method, the desired protein purification is essential to remove bacterial residue and other toxins. Hence, this technology is currently limited to nasal vaccine and injection formulation.

### 3.2. The Present Situation of Vaccines Produced by Plant Biotechnology That Target Respiratory Disease

For respiratory disease, many plant-bases vaccine candidates are available for a disease like *Bursal disease virus*, influenza, *Respiratory syncytial virus*, *Streptococcus pneumoniae*, *Bacillus anthracis*, *Mycobacterium tuberculosis*, and asthma [84]. These vaccines are safe and can be generated at a low cost by using low-cost bioreactors. It can be administered orally; hence, no antigen purification is needed, saving considerable production costs.

A plant-based vaccine against infectious *Bursal disease virus* used transient expression of *VP2* in *Nicotiana benthamiana* [85]. Plant-based vaccine for influenza used haemagglutinin (a surface glycoprotein that is involved in influenza virus infection) and M1 protein (most abundant structural matrix protein in the viral core) [86,87,88]. A pioneering study in the plant by D’Aoust et al. [88] reported the production of enveloped influenza VLPs. It opened the path for the large-scale production of a VPL-based plant-based vaccine for H5N1 influenza with a potential yield of up to 1500 doses per kg of infiltrated leaves [88,89]. Another study reported the formation of VLPs by expression of HAs from the strains A/Indonesia/5/05 (H5N1) or A/New Caledonia/7/2009 (H1N1). They were transiently expressed in *N. benthamiana* [90].

Another study reported enhanced immunogenicity of recombinant HA in an enveloped VLP over soluble antigen [91]. Further studies expressed different, HA antigens from A/Brisbane/59/07 [HAB1 (H1)], A/Brisbane/10/07 [HAB1 (H3)], B/Florida/4/06 [HAF1 (B)], and A/California/04/09 [HAC1], respectively) transiently in *N. benthamiana*. 400–1300 mg protein obtained from 1 Kg of fresh infiltrated leaf tissue [92]. Another study reported good immunogenicity and safety profiles of HAC1 and HAI-05 in animal pre-clinical studies [93].

Clinic trials of the HAC1 vaccine for the H1N1 virus were safe and well tolerable with mild adverse events compared to placebo. This vaccine was also immunogenic with the highest seroconversion rates based on virus microneutralization antibody titers and serum hemagglutination-inhibition [94]. 

One of the studies used a combination of a silica nanoparticle-based (SiO_2_) drug delivery system with a plant-produced H1N1 influenza hemagglutinin antigen (HAC1) and the mucosal adjuvant candidate bis-(3’,5’)-cyclic dimeric guanosine monophosphate (c-di-GMP). This vaccine induces systemic humoral immune responses in intratracheally vaccinated mice [95].

The *respiratory syncytial virus* causes illness in the lower respiratory tract in adults and children [96,97,98,99]. Recently, expressing the RSV fusion (F) protein gene in transgenic tomato plants, a fruit-based edible subunit vaccine against RSV was developed. In ripening tomato fruit, the F-gene was expressed under the control of the fruit-specific E8 promoter. Ripe transgenic tomato fruit orally administered to mice led to the induction of mucosal and serum RSV-F specific antibodies [100].

Diseases caused by *Streptococcus pneumoniae* (the pneumococcus), *Haemophilus influenzae,* and *Neisseria meningitidis* are responsible for almost two million deaths each year the children are under five years old [101,102]. Disease caused by *S. pneumoniae* remains high despite the extensive use of pneumococcal vaccines. It is mainly due to the absence of serotypes in the vaccine [103]. A recent study reported that plants could be engineered to synthesize bacterial polysaccharides, and these polysaccharides can provide protective immunity. They also demonstrated this principle using the serotype 3 capsular polysaccharide (a frequently isolated serotype from disease cases) of *S. pneumonia* [103]. Mice that are immunized with the extracts from recombinant plants were performed better with a lethal dose of pneumococci in a pneumonia mouse model, and the immunized mice display significantly elevated antibodies of serum anti-pneumococcal polysaccharide. This study provides evidence that plant biotechnology tools can successfully synthesize bacterial polysaccharides, and the recombinant polysaccharides produced from them could be used as potential vaccine candidates to protect against life-threatening respiratory infections [103].

Anthrax is another disease for which plant-based vaccines were effective. A Gram-positive bacterium, *Bacillus anthracis*, causes anthrax. Its spores remain viable even in the extreme environment for centuries. Within the host cells, these spores produce three-component anthrax toxins: edema factor (EF), lethal factor (LF), and protective antigen (PA) [104]. Inhalation of spores leads to *B. anthracis* via the respiratory tract leads to severe respiratory distress causing cyanosis, shock, and death [105]. Many studies related to the heterologous expression systems, including bacterial, viral, or plant systems, have been reported for vaccines [106,107,108,109]. Due to their natural bio-encapsulation protection from digestive enzymes, plant-based vaccines improve immune response in the gut system by gradually releasing the antigen [110,111]. PA is the main virulence factor to cause anthrax. Expression of PA in tobacco and tomato generates lethal toxin neutralizing antibodies in a murine model by intraperitoneal immunization [112,113]. Recently PA has been expressed in mustard by *Agrobacterium*-mediated transformation since mustard is commonly used as a stem and leaf vegetable and fodder meant for cattle in various parts of the world. In orally immunized groups, a specific mucosal immune response was observed.

Furthermore, in-vitro lethal toxin neutralizing potential indicated by the antibodies conferred in-vivo protection against toxin challenge. The immunoprotective response was observed in mice during oral immunization [114]. They use agroinfiltration plant transient expression systems for engineered, expressed, purified, and characterized full-length PA (pp-PA83) in tobacco plants. Immunization with these vaccines protected all the rabbits from the lethal aerosolized *B. anthracis*. The vaccine antigen formulated with Alhydrogel retained immunogenicity even after two-week storage at 4 °C and was stable (essential for clinical use) [115]. Anthrax protective antigen (PA-D4) domain-4 epitope has a vital role in enhancing protective immunity against virulent *B. anthracis*. One study successfully reported a recombinant protein that comprised the antigenic PA-D4 integration into the *c/e1* loop of HBcAg in transgenic tobacco. Plant-derived purified HB/PA-D4 protein injected into mice, and its sera display significant anti-HBcAg and PA-specific IgG titers [116].

Plant biotechnology-based vaccines are also made to prevent the infectious disease tuberculosis. *Mycobacterium tuberculosis* causes tuberculosis [117]. It can transmit from human to human via droplets expelled into the air via an infectious person. Death caused by TB even exceeded HIV, making it a more significant epidemic than expected. To date, seven oral plant biotechnology-based TB vaccines have been extensively evaluated either in experimental or pre-clinical and phase I clinical trials [117]. In Potato, Ag85B, ESAT-6, MPT64, and MPT83 antigens are expressed [118]; in tobacco, Acr, and Ag85B antigens are expressed [119]; in *Arabidopsis thaliana*, ESAT-6 fused to LTB and antigens are expressed [120,121]; in carrot CFP10 and ESAT-6 antigens are expressed [122]; and in lettuce and tobacco, Mtb72F (Mtb32/Mtb39) and ESAT-6 fused to CTB and its antigens expression in the chloroplast [123] (Table 4).

Asthma is also a chronic inflammatory disorder, where a plant-based vaccine is effective. Asthma affects about 300 million people worldwide. It is estimated that by 2025 asthma will affect an additional 100 million people [126]. In one study, a genetically modified narrow-leaf lupin (*Lupinus angustifolius* L.) expressing a potential allergen (sunflower seed albumin) (SSA-lupin) gene was examined whether it can suppress the development of asthma. The result indicated that SSA-lupin consumption promoted an Ag-specific IgG2a Ab response via CD4^+^CD45RB^low^ T Cell and IFN-γ -dependent mechanism [124].

In another study, transgenic Tg rice plants express in their seeds a fragment (residues 45–145) of Der p 1 containing the significant human and mouse T-cell epitopes. Oral administration of the Tg rice seeds to mice inhibits the allergen-specific IgE responses and allergen-specific T helper 2 (Th2) cytokine synthesis (IL-4, IL-5, and IL-13). This induction of oral tolerance was linked with inhibition of bronchial hyper-responsiveness (BHR) [125]. In tobacco leaves, the recombinant chimeric allergen R8 was successfully expressed. In the herbaceous leaf extracts, a pro-peptide was observed. This protein displays properties the same as tobacco with respect to IgE immune reactivity or the parental allergen ProDer f 1 that is expressed in *Escherichia coli* [127].

Since SARS-CoV2 is also a respiratory disease, developing a new plant-based vaccine study mentioned above can significantly impact it. There are already some applications by Medicago Inc., using the same virus-like particle platform, which it has used for a plant-based vaccine for H5N1 influenza in the study mentioned earlier.

Plant biotechnology-based vaccines are becoming a reality, even though their progress has been slower than expected. It is particularly true in oral vaccines, having the main drawbacks of poor reproducibility, a question mark in antigen stability, and bioavailability [128,129].

**Figure 2:**
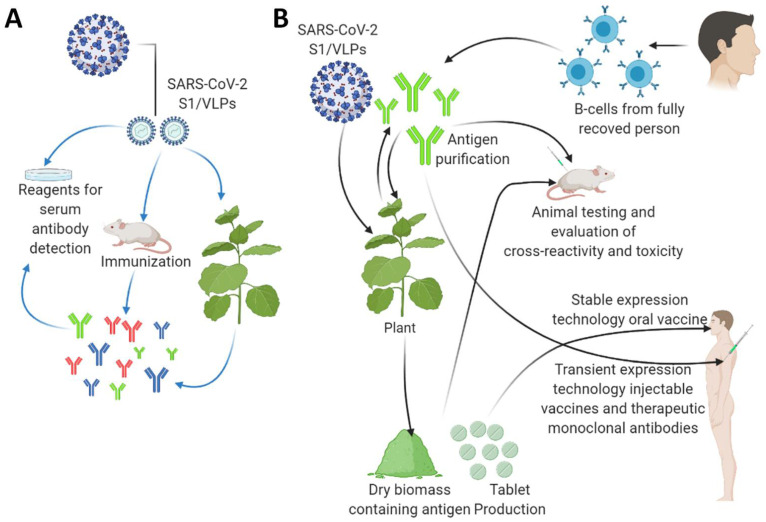
The applications of plant biotechnology-based production of diagnostic reagents and vaccine candidates against the SARS-CoV2. (**A**) Plant-based production of diagnostic reagents indicated by blue arrows. (**B**) Plant-based production of vaccine candidates against the SARS-CoV2 indicated by black arrows. A tobacco plant is shown as a model plant for both transient expression and stably transformed transgenic plants as a large-scale production platform. Genetic engineering approaches express target antigens by either stably or transiently transformation, enabling scientists to use different immunization approaches. The transient transformation method enables high antigen protein yields in the transformed plants purified to obtain injectable vaccines or therapeutic monoclonal antibodies. In a stable genetic transformation method, the edible plant species can provide oral vaccine formulations such as; capsules or tables with antigens from freeze-dried leaves. They can also be applied as a boosting agent. This figure is prepared by using Biorender (https://biorender.com/, accessed on 12 June 2021).

Plant biotechnology allows foreign protein expression in plants and projects a short-term approach for a potential vaccine candidate for SARS-CoV-2. The method of this expression will depend upon the nature of the targeted antigen. In the following section, we have discussed the idea of using a plant biotechnology bases platform as a possible approach for SARS-CoV-2 vaccine development (Figure 2).

## 4. Scope of SARS-CoV-2 Vaccine Development Using Plant Biotechnology Platform

Nanoparticles (NPs) and virus-like particles (VLPs) are the protein structures that have similarities with native viruses but do not contain a viral genome nor have any infectious ability, thus creating a safer platform for vaccine candidates [130]. Both NPs and VLPs constitute self-assembling proteins that display the epitope of interest at a higher density at their surface. Nanoparticles must have the antigenic epitopes repetitive and so that the innate humoral immune system and B cells are activated [131,132,133]. NPs/VLPs support antigen uptake by antigen-presenting cells (APCs), enhancing the immune system’s adaptive arms [134]. In the 21st century, many platforms for NPs/VLPs design have been evolved, including the usage of site-specific ligations-driven covalent links of individual folded proteins, viral core proteins, and non-covalent intramolecular formation of de novo protein nanostructure via intermolecular interactions. Both self-assembled protein NPs and VLPs offer highly stable, ordered, and monodisperse vaccine formulations and upscale production with bio farming. For the new vaccine development, NPs/VLPs are currently recognized as the most studied promising molecular carriers [130]. To develop VLPs Medicago Inc. (Quebec City, QC, Canada) used the *Nicotiana benthamiana* plant [135]. Medicago’s plant-derived COVID-19 vaccine candidate along with GlaxoSmithKline’s (GSK) pandemic adjuvant have entered into the phase 2/3 clinical trials. Medicago’s plant-derived vaccine candidate against COVID-19 uses Coronavirus-Like-Particle (CoVLP) technology in which vaccine composed of recombinant spike (S) glycoprotein and is expressed as virus-like-particles (VLPs). It is co-administered with GSK’s adjuvant. Two doses of 3.75 micrograms of CoVLP are administered 21 days apart. Data shows that the combination of the vaccine candidate and GSK’s pandemic adjuvant-induced a significant humoral immune response after two doses. Similar antibody responses were observed in younger and middle-aged adults, as well as elderly adults. (https://www.medicago.com/en/newsroom/; https://ir.ibioinc.com/press-releases; https://news.cision.com/expres2ion-biotechnologies, accessed on 12 June 2021) [136]. Kentucky BioProcessing, Inc. (KBP) (formally known as Large Scale Biology Corp.) candidate vaccine, COVID-19 Subunit Vaccine KBP-201, is in the 2nd phase of clinical trials. They have used *Nicotiana benthamiana* as a host plant/expression system. Both candidate vaccines have two doses scheduled after 21 days gap. They can be administered via the intramuscular route (Table 5). There are four other candidate vaccines from iBio, Inc. (New York, NY, USA), Akdeniz University (Turkey), Shiraz University (Iran), and Baiya Phyto-pharm/Chula Vaccine Research Center (Thailand) that are in the pre-clinical stage and have used the plant as an expression system. Many reports explain the role of NPs in SARS-CoV-2 in detail [136,137,138,139,140,141,142].

For the production of VLPs, several studies target Poliovirus, hepatitis B virus, human papillomavirus, influenza virus, Norwalk virus, human immunodeficiency retrovirus 1, rift valley fever virus, and foot and mouth disease virus [132,146,147,148,149,150,151,152,153,154,155,156,157,158]. Earlier experience of forming VLPs for MERS and SARS-CoV-1 antigens heterologous expressed in recombinant systems provides us the best platform for developing a vaccine against SARS-CoV-2. A study reported that in morphology, developed VLPs were similar to the virions of SARS-CoV-1. Another report stated that envelope proteins (E) and membrane (M) are sufficient enough for the efficient formation of virus-like particles, and they could be visualized by electron microscopy [159]. VLPs formed by membrane proteins of different origins activated immature dendritic cells (DCs) and enhanced the secretion of cytokines and co-stimulatory molecules’ expression [160]. 

Mucosal routes have emerged as attractive and promising routes for the vaccination of respiratory diseases. Mucosal immune response for VLPs is an essential aspect of vaccine success. In one study, mice were immunized with VLPs plus cytosine–phosphate guanosine (CpG) and VLPs intranasally. Both of them induced IgG specified to SARS-CoV1 [161].

Given that HA protein expression in plant-made VLPs vaccine successful, similarly, it is believed that for the development of SARS-CoV-2 VLPs, S protein expression might be necessary. Considering this, targeting the trans-Golgi secretion route by nuclear expression might yield a protein that, via secretion and glycosylation process, can produce VLPs for SARS-CoV-2 [162]. Forty-seven plant-based candidate VLPs vaccine has been developed for a wide range of disorders [163].

## 5. Concluding Remarks and Future Direction

There are a few risks that might be involved with the plant biotechnology platform-based vaccine [164]. Their risks include (1) Oral tolerance; if the antigen is delivered too frequently, the mucosal immune system becomes desensitized to the candidate vaccine, and the vaccination might no longer tackle susceptibility to the target disease. (2) Allergenicity; compared to the natural pathogen in plants, a transgenic product might be undergone by different post-translational modifications, which might induce new allergenic responses in the host during vaccination. Along with this, the use of oral adjuvants for mucosal linings stimulation might induce hypersensitive responses to other food proteins [165,166]. (3) Gene transfer; transfer of the antigen to the conventional food supply through genetic engineering could lead to oral tolerance. (4) Detrimental effects on the environment; natural loss and degradation of a gene during selection within the environmental system. The transgene is randomly inserted into the genome during gene transfer, which can lead to positional effects. These events make expression levels unpredictable, and the loss of endogenous genes is also a possibility that might leads to toxicity or allergenicity implications. Advancements in technologies can solve these limitations by providing alternative methods to achieve site-directed mutation through many mechanisms [78]. (5) Inconsistent dosage; an insufficient amount of antigen might not produce the desired immune response needed to protect against the deadly disease. Incorrect frequency or wrong dosage could lead to tolerance and reduce vaccine effectiveness in some candidates [167,168]. To overcome this limitation, proper clinical trials in animals and humans must determine the doses to generate a proper immune response. 

COVID-19 outbreak led to a global health emergency that demands new vaccines to cope with this pandemic. Plant biotechnology-based vaccine candidates offer an alluring approach for containing this virus. The available expression platform offers relevant directions for developing a candidate vaccine for COVID-19. The deconstructed viral vectors transient expression system is one of the alternative approaches for vaccine production where the tobacco as a host plant will allow for fast exploitation of plants as efficient large-scale biofactories for injectable vaccine candidates. A major disadvantage of this strategy is the potential loss of exogenous genes and ultimately loss of systemic infectivity. However, this can be prevented by using a subgenomic promoter derived from a different virus. It will lead to heterologous genetic recombination. Currently, six front-runner plant-based vaccines are based on this platform (Table 5). VLPs vaccine is another alternative option that provides an attractive approach for producing safe and efficient vaccines, which lacks replicative capacity, preserve antigenic determinant, and have high immunogenicity. VLPs based vaccines platform cannot be used for all types of viruses, which might be a major drawback for this technology. However, if its advantage is taken into consideration, the VLPs vaccine has vast potential. VLPs platform already has a proven track record in the case of earlier SARS-CoV-1. Hence, VLPs development based on different SARS-CoV-2 structural proteins is an excellent approach against COVID-19. Another approach is to develop vaccines based on edible plant species that are transformed at the nuclear level and administered as oral vaccines. It will provide mucosal immunity.

In 2020, the market size of global plant-based vaccines was estimated to be valued at 927.0 million USD, and in the next six years, it is expected to witness a growth rate of over 11.7%. Existing key players and new entrants in the plant-based vaccines market now focus on extensive clinical trial studies to develop plant-based vaccines for numerous therapeutic applications, including the COVID-19 vaccine. For example, Medicago Inc., a clinical-stage Canadian biotechnology company, uses plant-based technologies to develop and produce many novel vaccines and antibodies by cultivating several tobacco plants (*Nicotiana tabacum*) at its Durham’s Research Triangle Park in North America. This facility will be used in the testing and large-scale production of the flu vaccine. To develop the flu vaccine, Medicago conducted phase 3 clinical trials in March 2018. It is expected to be launched in the market soon during influenza season.

It requires almost five to six weeks to produce a plant-based vaccine compared to a five to six-month period preparing the vaccine in chicken eggs, which the various vaccine manufacturers are currently practicing. Along with these developments, monoclonal antibody production in plants can also provide another alternative plasma transfusion strategy. Antibodies developed in plants will be affordable and have safer intravenous treatment for critically ill patients (Figure 2). There are already approved plant-based vaccines for influenza that give hope to the potential of plant-based anti- COVID-19 vaccine. The Coalition for Epidemic Preparedness Innovations (CEPI) estimated that global vaccine manufacturing capacity would be only 2–4 billion doses annually, and by 2023–2024, not enough vaccines can be manufactured to meet global demands. This capacity might also be product-specific, along with some limitations: for example, whole-inactivated virus vaccines must be manufactured in a facility with biosafety level 3-capability. In addition to this administrative, the regulatory process of licensing, technology transfer, and the scale-up of vaccine manufacturing, purification or formulation might be time-consuming, and fulfilling these requirements in a time-bound manner will remain challenging. A plant-based vaccine platform can fill the gap and help maintain the demand/supply ratio. The coming years will be crucial to see the real potential of a plant-based vaccine for COVID-19 or any other pandemic.

## Figures and Tables

**Table 1:** The COVID-19 candidate vaccine in clinical trials.

	Platform	Candidate Vaccines	
		**Number**	**Percentage**
**1**	Protein subunit	32	Thirty-one
**2**	Viral Vector (non-replicating) (VVnr)	16	Sixteen
**3**	DNA	10	Ten
**4**	Inactivated Virus (IV)	16	Sixteen
**5**	RNA	16	Sixteen
**6**	Viral Vector (replicating) (VVr)	2	Two
**7**	Virus-Like Particle (VLP)	5	Five
**8**	VVr + Antigen Presenting Cell (VVr+APC)	2	Two
**9**	Live Attenuated Virus (LAV)	2	Two
**10**	VVnr + Antigen Presenting Cell (VVnr+APC)	1	One
**Total**		102	

**Table 2:** WHO list of candidate vaccines for COVID-19 in advanced trials [3].

Sr. No.	Vaccine Platform	Type of Candidate Vaccine	No. of Doses	Adjuvant	Schedule	Route of Administration	Developers	Phase	Clinical Trials (gov.Identifier)
1	Inactivated virus (IV)	CoronaVac; SARS-CoV-2 vaccine (inactivated)	2	Aluminium hydroxide gel (Algel)	Day 0 + 14	IM	Sinovac Research and Development Co., Ltd.	Phase 4	NCT04775069
2	Inactivated virus (IV)	Inactivated SARS-CoV-2 vaccine (Vero cell)	2	Aluminium hydroxide gel (Algel)	Day 0 + 21	IM	Sinopharm + China National Biotec Group Co + Wuhan Institute of Biological Products	Phase 3	NCT04612972
3	Inactivated virus (IV)	BBIBP-CorV, Inactivated SARS-CoV-2 vaccine (Vero cell)	2	Aluminium hydroxide gel (Algel)	Day 0 + 21	IM	Sinopharm + China National Biotec Group Co + Beijing Institute of Biological Products	Phase 3	NCT04510207 *
4	Whole-Virion Inactivated SARS-CoV-2 Vaccine (BBV152)	Inactivated virus vaccine	2	Aluminium hydroxide gel (Algel)	Day 0 + 14	IM	Bharat Biotech International Limited	Phase 3	NCT04641481; CTRI/2020/11/028976
5	SARS-CoV-2 vaccine (vero cells)	Inactivated virus vaccine	2	Aluminium hydroxide gel (Algel)	Day 0 + 28	IM	Institute of Medical Biology + Chinese Academy of Medical Sciences	Phase 3	NCT04659239
6	QazCovid-in^®^ -COVID-19 (Inactivated virus)	Inactivated virus vaccine	2	No	Day 0 + 21	IM	Research Institute for Biological Safety Problems, Rep of Kazakhstan	Phase 3	NCT04691908
7	Viral vector (Non-replicating) (VVnr)	ChAdOx1-S- (AZD1222) (Covishield, Vaxzevria)	1-2	No	Day 0 + 28	IM	AstraZeneca + University of Oxford	Phase 4	NCT04775069
8	Viral vector (Non-replicating) (VVnr)	Recombinant novel coronavirus vaccine (Adenovirus type 5 vector)	1	No	Day 0	IM	CanSino Biological Inc./Beijing Institute of Biotechnology	Phase 4	NCT04540419
9	Viral vector (Non-replicating) (VVnr)	Gam-COVID-Vac Adeno-based (rAd26-S+rAd5-S)	2	No	Day 0 + 21	IM	Gamaleya Research Institute; Health Ministry of the Russian Federation	Phase 3	NCT04741061
10	Viral vector (Non-replicating) (VVnr)	Ad26.COV2.S	1-2	aluminum phosphate adjuvant (Adjuphos)	Day 0 or Day 0 +56	IM	Janssen Pharmaceutical	Phase 3	NCT04614948
11	Protein subunit	SARS-CoV-2 rS/Matrix M1-Adjuvant (Full length recombinant SARS CoV-2 glycoprotein nanoparticle vaccine adjuvanted with Matrix M)	2	Matrix-M™	Day 0 + 21	IM	Novavax	Phase 3	NCT04583995
12	Protein subunit	Recombinant SARS-CoV-2 vaccine (CHO Cell)	2-3	Aluminium hydroxide gel (Algel)	Day 0 + 28 or Day 0 + 28 + 56	IM	Anhui Zhifei Longcom Biopharmaceutical + Institute of Microbiology, Chinese Academy of Sciences	Phase 3	NCT04646590
13	Protein subunit	VAT00002: SARS-CoV-2 vaccine formulation 1 with adjuvant 1 (S protein (baculovirus production)	2	AS03	Day 0 + 21	IM	Sanofi Pasteur + GSK	Phase 3	PACTR202011523101903 **
14	Protein subunit (SOBERANA 02)	FINLAY-FR-2 anti-SARS-CoV-2 Vaccine (RBD chemically conjugated to tetanus toxoid plus adjuvant)	2	Aluminium hydroxide gel (Algel)	Day 0 + 28	IM	Instituto Finlay de Vacunas	Phase 3	RPCEC00000354
15	Protein subunit	EpiVacCorona (EpiVacCorona vaccine based on peptide antigens for the prevention of COVID-19)	2	Aluminium hydroxide gel (Algel)	Day 0 + 21	IM	Federal Budgetary Research Institution State Research Center of Virology and Biotechnology “Vector”	Phase 3	NCT04780035
16	RNA based vaccine	mRNA -1273	2	No	Day 0 + 28	IM	Moderna + National Institute of Allergy and Infectious Diseases (NIAID)	Phase 4	NCT04760132
17	RNA based vaccine	BNT162 (3 LNP-mRNAs), Comirnaty	2	No	Day 0 + 21	IM	Pfizer/BioNTech + Fosun Pharma	Phase 4	NCT04775069
18	RNA based vaccine	CVnCoV Vaccine	2	CV8102	Day 0 + 28	IM	CureVac AG	Phase 3	NCT04674189
19	DNA based vaccine (ZyCoV-D)	nCov vaccine	3	No	Day 0 + 28 + 56	ID	Zydus Cadila	Phase 3	CTRI/2020/07/026352

IM = intramuscular; ID = intradermal. * This phase 3 trial assesses both the Wuhan (NCT04612972) and Beijing (NCT04510207) vaccine in the same study. ** Pending confirmation on the phase of the study, which is not specified in the registry.

**Table 3:** Summary of the different expression approaches for producing plant-based vaccines and their function as MERS/SARS-CoV-1 vaccines.

Method	Features	Limitations	Target/Plant Species	The Protein Used/Route of Inoculation	Experimental Phase	Dose	Degree and Type of Protection Generated	Functions	Reference
**Stable nuclear transformation**	Seed bank possible; Inheritable antigen production; Many methods are available for different crops	Random insertion; Possibility of horizontal gene transfer; position effects and gene silencing; transformation is tedious	Full and truncated S protein/tomato and tobacco	Purified Protein/In saline and oral immunization	Pre-clinical	500 mg of dry tomato fruit, 50 mg of dry tobacco root, 2-week intervals, after a 4-week booster dose of 1 μg of commercially obtained S peptide without adjuvant.	Significantly increased levels of SARS-CoV-specific IgA after oral ingestion of tomato fruits expressing S1 protein.	Expression of SARS-CoV S protein (S1) in tomato and tobacco plants and after oral ingestion of tomato fruits, mice display elevated SARS-CoV-specific IgA levels.	[73]
**Transient nuclear transformation**	High and rapid protein production; Industrial scale production	The seed bank is impossible; requires purification of the antigen;	Partial spike protein of SARS-CoV; recombinant nucleocapsid (rN)and the membrane protein (M)/tobacco	Purified Protein/Intraperitoneally	Pre-clinical	2–4 μg rN protein	Vaccination of BALB/c mice with tobacco-expressed rN protein successfully led to a specific B-cell response.	Produced S1 proteins in chloroplast- and nuclear-transformed plants display potential in safe oral recombinant subunit vaccine. The expression of IL-10 and IFN-γ was up-regulated during the vaccination of rN protein, while IL-4 and IL-2 expression were not.	[74,75,76]
**Transplastomic technologies**	Multigene expression Highly productive; Better biosafety; site-specific insertion via recombination; Unaffected by silencing or position effects	Lacks complex post-translational modifications; Limited protocols available for limited species; generation of lines are tedious	N-terminal fragment of SARS-CoV S spike protein (S1)/Tomato and tobacco	Purified Protein/In saline and oral immunization	Pre-clinical	500 mg of dry tomato fruit, 50 mg of dry tobacco root, 2-week intervals	The mice parenterally primed with plant-derived antigen developed an immune response after booster immunization.	Sera of mice display the SARS-CoV-specific IgG.	[73,77]

**Table 4:** Plant-based vaccines against respiratory disease.

Vaccine Candidate	Plant	Antigen	Animal	Route of Inoculation/Doses	Degree of Protection	Immunological Data	Reference
**Bursal disease virus**	Tobacco	VP2/extracted	Embryonated eggs of White Leghorn chickens	Intramuscular/12 μg of VP2 and equal volume of Freund’s adjuvant and 1% total volume of Tween 40	Plant-derived VP2 elicited an antibody response with neutralizing activity	VP2 produced in plants can elicit an appropriate immune response in chickens	[85]
**Respiratory syncytial virus**	Tomato	F-gene/extracted	BALB/c mice	Oral immunization/each mouse was given 5–7 g of ripe tomato fruit containing recombinant RSV-F protein and consumed 3–4 g.	Transgenic-fruit-derived RSV-F antigen primed a mixed type 1–2 T-helper cell immune response and further that this RSV-boost-induced response showed some bias towards the Th1-type	Ripe transgenic tomato administered to mice orally that led to the elevation of mucosal and serum RSV-F specific antibodies	[100]
***Streptococcus pneumoniae***	Tobacco	Serotype 3 capsular polysaccharide/extracted	MF1 female mice	Intraperitonea l/2 µg plant-derived pneumococcal polysaccharide per mouse in 67 µL PBS and 33 µL Inject alum adjuvant (Pierce, Rockford, IL, USA)	None of the fifteen animals given wild-type extract were alive ten days after the challenge, whereas eight of the fourteen immunized with transgenic extract survived	Immunized mice had significantly elevated levels of serum anti-pneumococcal polysaccharide antibodies.	[103]
***Bacillus anthracis***	Tobacco, Tomato, and Mustard	Protective antigen (PA)/extracted	BALB/c mice	Intraperitoneal/Protein extracted from tomato leaves was mixed with complete Freund’s adjuvant (for the first dose) and incomplete Freund’s adjuvant (for subsequent doses) in a ratio of 1:1.	The PA expressed in nuclear transgenic tomato plants was able to generate an antibody-mediated immune response.	A specific mucosal immune response was observed	[112,113]
***Mycobacterium tuberculosis***	Potato, Tobacco, Carrot, Arabidopsis, and Lettuce	Ag85B, ESAT-6, MPT64, MPT83, Acr, Ag85B, ESAT-6 fused to LTB, CFP10, ESAT-6, Mtb72F, and ESAT-6 fused to CTB/extracted	C57BL/6 mice, BALB/c mice, Female ICR mice, *Seryi velikan* strain rabbits.	Orally, intranasal, intraperitoneal/BCG group were fed orally with 1.8 × 10^7^ CFU BCG in 100 μL saline per mouse. The mice of the combined-plant vaccine group were fed with 1ml of the concentrated transgenic potato extract. Mice were immunized subcutaneously with 100 μL of BCG administered at the base of the tail or with 10 μg TB-RICs preparation (in 30 μL) intranasal, under isoflurane anesthesia. Test animals were provided with 3 g of the mix (92.6 μg of plant-made LTB-ESAT-6). Feed treatments were given on days 0, 7, 14, and 28.	Generating antigen-specific, Th1 response	Antigens expression	[118,119,120,121,122,123]
**Asthma**	Lupin	SSA-lupin/extracted	BALB/c mice	Intraperitoneal/50 μg of SSA or OVA in alum (1 mg/mL) dissolved in PBS (final volume 200 μL). On days 14 and 16. B, Lupin, and SSA-lupin induced systemic sensitization and DTH responses.	GM plant-based vaccine can promote a protective immune response and attenuate experimental asthma	Consumption of SSA-lupin promoted the elevation of an Ag-specific IgG2a Ab response through CD4+CD45RBlow T Cell and IFN-γ -dependent mechanism	[124]
**Bronchial hyper-responsiveness**	Rice	Der p 1/purified	BALB/c mice	Orally vaccinated by feeding 6–8-week-old female BALB/c mice were orally vaccinated by feeding 0.5 or 5 mg purified recombinant Der P1 dissolved in PBS on day 1. Mice were given four intraperitoneal injections of 2 μg of recombinant Der p 1 adsorbed to alum adjuvant.	Prophylactic efficacy of oral vaccination with Tg rice seeds accumulated Der p 1 (45–145) in a mouse model of asthma, reducing allergic airway inflammation and reduced BHR.	Oral administration of the Tg rice seeds to mice inhibits the allergen-specific IgE responses and allergen-specific T helper 2 (Th2) cytokine synthesis (IL-4, IL-5, and IL-13)	[125]

**Table 5:** Current status of WHO listed plant-based vaccine candidates for COVID-19 under trial stages.

Vaccine	Vaccine Platform Description	Developers	Transformation Method	Expression System	Status	No. of Doses	Schedule	Route of Administration	References
COVID-19 VPL Vaccine (CoVLP)	Virus-like particle (VLP)/Spike protein	Medicago Inc. (Québec, Canada)	VLPExpress™ system (Agro-infiltration)	*Nicotiana benthamiana*	Phase 2/3	2	Day 0 + 21	IM	[135,143]
COVID-19 Subunit Vaccine (KBP-201)	Protein Subunit	Kentucky BioProcessing, Inc. (KBP)	Agro-infiltration	*Nicotiana benthamiana*	Phase 2	2	Day 0 + 21	IM	[144]
COVID-19 Subunit Vaccine (IBIO-201)	Protein Subunit/Spike protein	iBio, Inc. (NY, USA)	FastPharming™ system (Agro-infiltration)	*Arabidopsis thaliana*	Pre-clinical	NA	NA	NA	[145]
COVID-19 Subunit Vaccine	Development of recombinant protein-based S1 and S2 (Spike) and nucleocapsid subunits vaccines using a plant expression vector.	Akdeniz University (Turkey)	Agro-infiltration	*Nicotiana benthamiana*	Pre-clinical	NA	NA	NA	[142]
COVID-19 VLP	Virus-like particle/Spike protein	Shiraz University (Iran)	Agro-infiltration	*Nicotiana benthamiana*	Pre-clinical	NA	NA	NA	[142]
COVID-19 Subunit Vaccine	Plant-based subunit (RBD-Fc + Adjuvant)/Spike protein	Baiya Phytopharm/Chula Vaccine Research Center (Thailand)	Agro-infiltration	*Nicotiana benthamiana*	Pre-clinical	NA	NA	NA	[3]

## Abbreviations

ACE2	Angiotensin Converting Enzyme 2.
APCs	Antigen Presenting Cells.
CEPI	Coalition for Epidemic Preparedness Innovations.
CoVLP	Coronavirus-Like-Particle.
CRISPR/Cas9	Clustered Regularly Interspaced Short Palindromic Repeats /CRISPR associated protein 9.
DNA	Deoxyribose Nucleic Acid.
EULs	Emergency Use Listing.
GSK	GlaxoSmithKline.
IV	Inactivated Virus.
KBP	Kentucky BioProcessing.
LAV	Live Attenuated Virus.
PS	Protein Subunit.
BHR	Bronchial Hyper-Responsiveness.
SARS-CoV-2	Severe Acute Respiratory Syndrome Coronavirus-2.
SSA	Sunflower Seed Albumin.
TALEN	Transcription activator-like effector nucleases.
UTRs	Untranslated Regions.
VLPs	Virus-Like Particles.
WHO	World Health Organization.
